# Becoming a dentist: faculty perceptions of student experiences with threshold concepts in a Canadian dental program

**Published:** 2018-11-12

**Authors:** Jacqueline Green, Kari Rasmussen

**Affiliations:** 1 Educational Research and Scholarship Unit, School of Dentistry, Faculty of Medicine and Dentistry, University of Alberta, Alberta, Canada

## Abstract

**Background:**

In each discipline, there are moments where students “get stuck” in their education and/or training and are often unable to move forward. These moments may be caused by threshold concepts as they represent a portal that students must cross in order to become successful in their chosen profession. This study investigated the threshold concepts from the instructors’ perspective that students must navigate as they transform from learners to dentists within a dental program.

**Methods:**

Two focus groups with faculty members within the School of Dentistry, University of Alberta were completed in the fall of 2017. Focus groups explored the faculty’s perception of the students’ transition from learner to dentist, difficult moments in the program, and the students’ ability to navigate the program successfully.

**Results:**

A qualitative phenomenographic analysis of the faculty focus group transcripts identified four potential threshold concepts within the dental program: 1) dealing with the whole patient, 2) accountability, 3) that you may not know everything, and 4) problem solving and adapting during practice.

**Conclusion:**

This study demonstrates that there are concepts within a dental program that faculty believe students must navigate in order to transition from learner to dentist. These concepts may inform curriculum design as well as other disciplines in the health sciences.

## Introduction

In each discipline, students come face to face with particularly difficult and important concepts. They may “get stuck,” unable to move forward in their chosen program until they can master these concepts. To be successful in their program, and later as a professional, students must demonstrate they have grasped these difficult concepts. Meyer and Land have termed these important and difficult concepts in a curriculum the threshold concepts, describing them as “akin to a portal, opening up a new and previously inaccessible way of thinking about something” (pg. 1).^1-4^

Since their inception, researchers have investigated threshold concepts within many disciplines including economics,^5^ engineering,^6^ geography,^7^ and leadership.,^8^ Two reviews have highlighted threshold concepts.^9,10^ Studies of threshold concepts in dental education have discussed them in general and attempted to define them specifically within dentristy.^11-14^ Our study examined threshold concepts required to become successful novice dentists upon completion of a Canadian dentistry program from the faculty perspective.

### Background

Meyer and Land^1-4^ have proposed that a threshold concept possesses the following characteristics:

- *transformative*, that is it may transform one’s perception of a subject or identity;
- *irreversible*, as once the new knowledge has been accepted it is unlikely to be forgotten or only unlearned with great difficulty;
- *integrative* in that it exposes the previously hidden interrelatedness of concepts;
- *bounded* in that conceptual space will have terminal boundaries, and may border with thresholds into new conceptual areas;
- troublesome in that it may represent what Perkins^15^ refers to as *troublesome knowledge*; that is knowledge that is alien, or may appear counter-intuitive, or even intellectually absurd at face value.

These characteristics distinguish threshold concepts from the core, or key, concepts of a particular discipline as these concepts do “not necessarily lead to a qualitatively different view of subject matter” (pg. 4).^1^ This transformative nature of threshold concepts is important for student learning as well as for curriculum development. A program built around the threshold concepts of a discipline may allow students to master these concepts, moving through the program more efficiently while supporting students through the educational landscape.^2^

As students move through their program, they are transformed by the threshold concepts. Arnold van Gennep has called those moments of intense growth “rites of passage.”^16^ He describes rites of passage as “the rites which accompany every change in place, state, social position, and age.”^16^ He proposed that these rites of passage have three phases: a separation phase, a liminal (or transitional) phase, and a re-integration phase.^16^ Victor Turner further defined the liminal phase as “betwixt-and-between,” where individuals are suspended, not belonging to either state of being.^17^ Students traverse through a particular threshold concept toward deeper understandings, attempting to leave previous (mis)understandings behind, but not yet fully able to comprehend the new concept.

The notion of threshold concepts as liminal spaces may be helpful for curriculum development. Threshold concepts become a mechanism for transformation.^18,19^ This shift may be sudden, in the form of an “aha” moment or protracted over a period of time. It may also involve oscillation between states.^19^ This transformation, and especially the oscillation between states, may be a source of anxiety for students as they attempt to determine where they fit in their program.^4^ Additionally, student acquisition of transformational knowledge may bring with it new and empowering forms of expression that characterize ways of disciplinary thinking; threshold concepts may transform students from learner to professional, enabling them to adopt the ways of thinking and practicing of their chosen field.^14^ It is this aspect of threshold concepts which give them their power; students may never be successful in their profession if they cannot learn to *think* like others in their occupation.

Understanding the particular threshold concepts both within a discipline, and during the transformation from student to professional, will inform the teaching practice of moments in the curriculum students may find particularly difficult. Land et al.^3^ define threshold concepts as “jewels in the curriculum”, key areas where students likely will encounter troublesome knowledge that they must master.

At the time of writing, the School of Dentistry at the University of Alberta was undergoing a curriculum renewal. The aim of the School is to create an experiential learning curriculum that focuses on improving the student experience within the program as well as restructuring the learning pathway and ensuring appropriate content is delivered to students. In addition, the School’s assessment philosophy will be restructured. As this renewal unfolds, research investigating the threshold concepts within the program is timely and allows for the creation of a curriculum that aims to support students’ transformation from a learner to an entry level practitioner. While there have been those who have advocated for threshold concepts to be used during curricula development, there are few studies that document the specific utilization of threshold concepts for this purpose. Therefore, it was the intention of the authors to utilize this study as a foundation to inform the curriculum renewal within the School of Dentistry at the University of Alberta.

## Methods

This research study was approved by the University of Alberta’s Human Ethics Review Board, Pro00074657. As a first step in determining the potential threshold concepts within the University of Alberta’s Doctor of Dental Surgery (DDS) program, we conducted two focus groups with faculty members in the dental program in the fall of 2017. The purpose of the focus groups was to capture the faculty members’ perspectives of the particular points in the curriculum that students found challenging as well as the students’ experience with potential threshold concepts within the dental program as part of their personal educational training. The focus group questions were created in order to explore faculty member’s conceptions of the students’ transition from learner to dentist, the difficult moments their students faced in the program, and the students’ ability to navigate through this transformational phase.

The School of Dentistry, which celebrated its 100-year anniversary in 2017, is housed within the Faculty of Medicine and Dentistry at the University of Alberta. Encompassing three buildings at the University, the School incorporates a research facility, a simulation lab, and a dental clinic. In addition to the DDS and Dental Hygiene programs, students may also attend the school for graduate degrees and specializations following completion of an undergraduate program. Each year there are 32 students accepted into the program, with up to 12 students added into an advanced placement program each year for those who have obtained certification for dental practice from a foreign institute. The program is structured such that students move from basic and medical sciences (pre-clinical), to laboratories that utilize mannequins to simulate clinical experiences, to the clinic where they work with patients.

### The participants

All faculty members involved in the DDS program were sent an email explaining the study and were asked to volunteer to participate in the focus groups. The faculty members emailed included full time and part time faculty who held clinical or academic appointments and taught students in a classroom or clinical environment, as well as foundational researchers in the department who taught and supervised dental students, and those involved with graduate studies. Fourteen faculty members expressed an interest in the study. We scheduled two focus groups to accommodate all potential participants. Four faculty members were unable to attend the sessions, leaving each focus group with five members in attendance. Those that attended were full-time faculty members, and included nine members with clinical academic appointments (of which many have taught both in classrooms and clinically) and one with a foundational science academic appointment. In addition, the focus groups included members from a variety of sub-disciplines within dentistry including orthodontics, endodontics, pediatric dentistry, integrated patient care, and oral medicine. We gave all participants in attendance at the focus group information sheets explaining the study and we asked them to read these and sign consent forms if they agreed to participate.

For a number of reasons we chose faculty members for this study as the first step in identifying potential threshold concepts within the dental program at the University of Alberta. As the faculty members are involved in the teaching of each year in the program, and have taught multiple cohorts, we believed that their participation in this study would add valuable insight into those moments in time that students have struggled in the program. In addition, the logistics of utilizing students as participants in a study investigating threshold concepts would be challenging for an introductory study, as there are multiple years and times within the program that students can be accessed. It was our intention to utilize faculty members within the program during the early phases of our research to explore their perceptions of the moments in time that students struggle in the dental program and if these moments in time are potential threshold concepts. We expect to include students in subsequent studies to determine if the threshold concepts described by faculty members resonate with students in the program.

### The focus groups

The researchers, two members of an Educational Research and Scholarship Unit within the School, completed the focus group interviews and subsequent analysis. This unit was established to support the scholarship of teaching and learning (SoTL) within the school and engage with faculty members to support educational research. As the researchers have been trained in research methodology, and are not clinicians, they employ a unique position in the School and are capable of acting as facilitators for research projects utilizing students and faculty members without the potential for a power dynamic.

Prior to beginning the focus group interviews, the facilitators explained the definition of threshold concepts according to Meyer and Land.^1-4^ As well, Perkins’ definition of troublesome knowledge was discussed.^15^ As many of the faculty members were unfamiliar with threshold concepts or troublesome knowledge, this ensured that each participant had some background information in order to identify potential threshold concepts within their particular course or experience as an instructor in the broader dental program. The definitions were then placed on boards and left up around the room so participants could access these descriptions throughout the session. Examples of threshold concepts found in various disciplines were also given to provide comparisons between key concepts, troublesome knowledge, and threshold concepts.

Each of the focus groups were audio recorded to produce verbatim transcripts for analysis. Analysis was completed utilizing a phenomenographic approach. Created in the 1970s by Ference Marton, Roger Saljo, Lars-Owe Dalgren, and Lennart Svensoon as a methodology to investigate learning, phenomenography is a qualitative research methodology which investigates how individuals experience a certain phenomenon.^20^ Ference Marton defines phenomenography as: “… a research method adapted for mapping the qualitatively different ways in which people experience, conceptualize, perceive, and understand various aspects of, and the phenomena in, the world around them.” (Pg. 31)^21^

As phenomenography is concerned with the qualitatively different ways people can experience a phenomenon, it is an appropriate approach for data collection and analysis investigating faculty perceptions of student experiences with potential threshold concepts in the dental program. The navigation through the challenges and liminal spaces of the dental program is a personal journey for students which have been witnessed by faculty over time. While the faculty members themselves do not experience these moments, they witness the students during this transformation and are able to describe their perceptions of the students’ experiences and the points in time that students may experience threshold concepts. Employing a phenomenographical approach in this way allows the potential threshold concepts experienced by students to be presented categorically, as they were described by those members of the faculty that participated in the research study.

Both researchers were involved in all aspects of the data analysis. Phenomenographic analysis is completed through a series of stages illustrated by Sjostrom and Dalgren.^22^ These stages include: 1) familiarization of material through reading the transcripts, 2) compilation of individual responses to a question regarding the data, 3) condensation of the responses to a find a central theme or dialogue, 4) grouping these similar themes together, 5) comparing of the categories to establish borders between each one, 6) naming of the categories, and 7) utilizing a contrastive comparison of the categories to produce descriptions for each.^22^ The result of this analysis is a description of the faculty members’ conceptions of potential threshold concepts within the dental program.

Following data analysis and presentation of the outcome space, each potential threshold concept was validated according to the aforementioned guidelines from Meyer and Land^1-4^ to ensure that those concepts faculty members felt were crucial to the dentistry program were indeed threshold concepts and not simply key concepts in a course or program. This was done by ensuring that each category presented by the faculty met each guideline described by Meyer and Land;^1-4^ any concepts that did not fit were not considered threshold concepts. For example, participants mentioned that students struggled with viewing 2D radiographs and translating information to patients in 3D. This concept is not transformative for students entering into dental practice, and thus was not considered a threshold concept.

## Results

Analysis of the focus group transcripts revealed four potential threshold concepts that faculty believe students must navigate in order to be successful in their transformation from learner to dentist.

### 1. Dealing with the whole patient

Faculty members described that students continually struggled with the concept of focusing on the whole patient in the program. Often faculty would state they felt the transformation from student to dentist happened when students understood that they had to take into account the medical history, the patient as an entire human being with multiple health concerns, and that they must learn to build relationships with their patients. Faculty felt that students were not able to transform from a learner into a dentist until they were able to understand they must care about the patient as a whole, including caring about their patients on a deeper rather than superficial level. Participant A (Focus Group 1) demonstrated that students must view the patient as a whole by stating: “But it is around treating the patient as a whole. … this is a person with a health history with medical concerns, medications they’re taking. It’s … pulling all of that together for the best interest of the patient….” In addition, participants felt that students must learn to pull all the disciplines they were taught together in order to effectively treat their patient. For example, Participant H (Focus Group 2) commented that “[T]hey start looking at this patient as a whole, and they tie all those disciplines together to treat that patient as a whole. And that’s when the transformation happens.”

### 2. Accountability

Participants within the focus group found that many students had difficulty grasping the concept that they will be independent dentists following graduation and therefore must be confident and accountable in their practice. Faculty members felt that to transform from a student to a dentist, one must accept responsibility for their patients while they are in their care. Many faculty members felt that responsibility for their practice, and patients, was a critical component in becoming a dentist as once they complete the program they are no longer able to defer this obligation to their instructors. Participant J (Focus Group 2) describes this concept when they remark, “… you may not have had this experience while you were a student, but it is up to you now. You are the end of the road.” In order to become accountable, participants stated that students must feel confident in their abilities. “That’s when they say, oh, I can - I can do it. I know myself, and I feel confident. So that’s - that’s what I have seen as a clinician” has been stated by Participant C (Focus Group 1).

### 3. You may not always know everything

Faculty members described that students in dentistry who have demonstrated high levels of accomplishment in order to successfully navigate the admissions process struggle with the notion that they may not always be right, or not always have an answer to everything, once they enter the DDS program. Participants in the focus group noted that students find it difficult to understand that dentistry as a discipline is about continual learning and that they will not always do a perfect job. This can be described by Participant B (Focus Group 1): “Because I can’t know everything. And I tell the students that. It’s okay not to know, but you need to know where to look to answer that why. … I always used to say I’m getting a practice - I’m getting a license to practice dentistry when I get out of here. Practice, practice getting better.” Participant A (Focus Group 1) also commented that “they’re afraid to learn some of the things, and to do some of what we ask them to do because they’re afraid to make a mistake.” In addition, many commented that students seek help from instructors and are unable, or unwilling, to investigate solutions to problems themselves. In fact, “they [Students] often go from instructor to instructor to get the answer they want.” was noted by Participant I (Focus Group 2). This includes the ability to self-reflect in order to ensure that work is accurately assessed once students leave the School and no longer have instructors evaluating work for them.

### 4. Problem solving and adapting

Faculty members commented that students have difficulty accepting that there may be more than one way to solve a problem presented by the patient and that students struggle synthesizing facts and linking previous knowledge to clinical experiences. Dentistry, as with many other disciplines, requires critical thinking in order to solve problems and decide the best mode of treatment for a particular patient. Focus Group participants felt that when there is variation in patients, or there may be multiple ways to complete a task, students are unsure how to adapt. Participant I (Focus Group 2) describes this when they state: “…not only your physical ability to do what you have to do, but your critical thinking in, you know, I have problem X and I can probably solve it five ways, but what’s best for this particular patient in their particular situation?” Many faculty members felt that if students better understood the decision making process, they may be able to adapt more effectively, enabling them to successfully transform from a learner to a novice dentist. “… You know, you get to a point where you need to make that transition between what you’re doing and why you’re doing it… and it’s a very important transition for professional health care folks, because once they understand the why, then that’s - that’s transformative”, as expressed by Participant D (Focus Group 1). This ensures that students know how to complete procedures or handle problems, but also why they would deal with situations in a certain way. In addition, students also have difficulty moving beyond procedural activities as the program is based on acquiring certain numbers of experiences; “… That transformation into a dentist doesn’t come until they don’t have to worry about a requirement or a grade.” (Participant H, Focus Group 2).

## Discussion

Students entering dental programs must acquire a certain amount of learning in order to graduate and become successful novice dentists. While much of this learning involves psychomotor skills, dental procedures, and patient care, this research study has demonstrated that there are additional concepts that must also be mastered in order to ensure accomplishment following graduation. Often when curriculum is designed only these concrete skills and facts are placed explicitly in the design, leading to missed expectations of the learner in terms of achieving a certain professional identity. This research study has identified four potential threshold concepts, as described by faculty members, which students must navigate in order to transform from a learner to a dentist. Faculty members felt that the students within the DDS program at the University of Alberta frequently struggled with these concepts, with many noting that they may not acquire these threshold concepts until late in the program, or following graduation.

These potential threshold concepts reflect topics that are not easily taught in a course. Thus, students who are very successful in their studies and have demonstrated high levels of intelligence may find they struggle to master these concepts irrespective of their grade in the program. As dentistry routinely involves patients presenting multiple and diverse problems, uncertainty, and critical thinking, it can be speculated that learning procedures and theory is not sufficient for students to successfully transform into dentists. Participant I in Focus Group 2 noted this when they commented “And I mean that totally applies to restoring, when you teach them to cut a crown prep a certain way or a filling a certain size. That’s what we taught you, but that’s probably not what you’re going to do on a person.” When considering the potential threshold concepts highlighted above, one can see how creating a curriculum focused on supporting students navigating these liminal spaces will allow for many to successfully and smoothly transform from learners into novice dentists. Courses that focus on professionalism and support students as they transition from learner to entry-level practitioner would provide assistance as they cross the liminal spaces of the threshold concepts. In addition, ensuring that instructors are aware of the threshold concepts in their courses may help them to recognize when students are struggling and create discourses that can address students’ anxieties with the liminal state of these concepts.

Utilizing the aforementioned threshold concepts for curriculum development in the School of Dentistry would allow the program, and specific courses, to be structured in order to facilitate student’s knowledge procurement. This can be done through the insertion of a discussion surrounding a particular threshold concept at a time where instructors have found students are struggling, or designing courses that specifically address these challenging points. One conceptual model places threshold concepts, student learning, and curriculum together as intersecting ideas.^23^ Instructors should think about each intersection and pose questions such as “what are the tacit aspects of the curriculum and/or discipline” (p. 540) to create a curriculum which integrates threshold concepts.^23^ Although we provide examples of our own personal experiences with threshold concepts, more research must be done to investigate how these concepts can influence student learning and curriculum design.

While this study has identified concepts believed to be threshold concepts for dental students, they may also fall within the broader scope of health professions. As these are not specific dental concepts, but rather concepts that make the transition to a dentist successful, they may also apply to other health professions such as nursing, medicine, and pharmacy. To investigate this fully, more work regarding different professional disciplines must be done. This would allow researchers to determine if threshold concepts in professionalism are shared among other disciplines beyond the health sciences.

### Study limitations

There are a number of limitations in this research study. The first limitation is that the focus groups may not have captured a representative example of the entire School of Dentistry faculty’s perception of the potential threshold concepts they believe students experience within the program. While the researchers attempted to include a representative example of the various types of faculty within the program, the participants were limited to those members of the faculty who agreed to attend, and thus some bias may have been introduced as a result. For example, there were no part time faculty present in the focus groups and therefore we cannot say with certainty that our focus groups were representative of the faculty members within the School. Secondly, many of the participants were focused on what they felt students were lacking in their training, and as such, there may be some threshold concepts missed, as there may be thresholds that students cross early in their training that faculty members were not attentive to. As this study aimed to investigate the threshold concepts experienced by students during the dental program, the focus group participants were concentrated upon moments they felt that students struggled, with some potentially not able to cross them until following their graduation. Further exploration of student perceptions of threshold concepts may aide in the resolution of this limitation. Thirdly, many of the participants were uncertain of the definition of a threshold concept, and how this may differ from a key or core concept. While efforts were made to ensure that faculty understood these definitions, the researchers note there may still have been some confusion which may lead to bias within the participants’ comments in the focus group.

### Conclusion

This research study was able to identify from a faculty perspective four potential threshold concepts students must navigate in order to become successful novice dentists following graduation from a dental program in Canada. While more work must be done with students to fully understand the threshold concepts within a dental program, results from this project have demonstrated that there are concepts which students struggle with in the dental program that are essential for success.

### Areas for future research

This research study was the first step in defining the threshold concepts within the DDS program at the University of Alberta. As such, more work must be done to investigate if there are other threshold concepts within the dental discipline, how these threshold concepts are navigated by students, and the impact of creating a curriculum in order to support students as they navigate through threshold concepts.

In order to fully understand how learners navigate the threshold concepts within a discipline, research including students as participants must be done. Further research with students would allow for a confirmation of the threshold concepts discussed in this work, and would allow for a more in-depth discussion of how the navigation of these threshold concepts impacts learning. In addition, research into the broader scope of dental programs in Canada and the world, and beyond into other disciplines, would allow for a more thorough understanding of the impact threshold concepts have on student learning and experience as well as curriculum design.
